# Supplementary material for: Topical miRNA Delivery via Elastic Liposomal Formulation: A Promising Genetic Therapy for Cutaneous Lupus Erythematosus (CLE)
Source: Int J Mol Sci. 2025 Mar 14;26(6):2641. doi: 10.3390/ijms26062641 (PMC11942213; doi:10.3390/ijms26062641)
Supplement: Supplementary file 1 [file ijms-26-02641-s001.zip › ijms-3428375-supplementary.pdf]

Supporting Information

Topical miRNA Delivery via Elastic Liposomal Formulation: A Promising Genetic Therapy for Cutaneous Lupus Erythematosus (CLE)

Blanca Joseph-Mullol [1], Maria Royo [1], Veronique Preat [2], Teresa Moliné [3], Berta Ferrer [3],

Gloria Aparicio [4], Josefina Cortés-Hernández [1,*] and Cristina Solé [1,*]

[1] Rheumatology Research Group, Lupus Unit, Hospital Universitari Vall d'Hebron, Institut de Recerca (VHIR), Universitat Autònoma de Barcelona, 08035 Barcelona, Spain; blanca.joseph@vhir.org (B.J.-M.); maria.royo@vhir.org (M.R.)

[2] Louvain Drug Research Institute—Advanced Drug Delivery and Biomaterial, Universite Catholique de Louvain, 1200 Brussels, Belgium; veronique.preat@uclouvain.be

[3] Department of Pathology, Hospital Universitari Vall d'Hebron, Institut de Recerca (VHIR), Universitat Autònoma de Barcelona, 08035 Barcelona, Spain; teresa.moline@vhir.org (T.M.); bferrer@vhebron.net (B.F.)

[4] Department of Dermatology, Hospital Universitari Vall d'Hebron, Institut de Recerca (VHIR), Universitat Autònoma de Barcelona, 08035 Barcelona, Spain; mariagloria.aparicio@vallhebron.cat

* Correspondence: fina.cortes@vhir.org (J.C.-H.); cristina.sole@vhir.org (C.S.); Tel.: +34-93-4894045 (C.S.)

INDEX

1. Supplementary Methods……………………………………………………………………………………..3
2. Supplementary Figures……………………………………………………………………………………...5
3. Supplementary Tables………………………………………………………………………………….….10

1. Supplementary Methods

Airyscan and Z-stack analysis

Live-cell imaging was performed on a Zeiss LSM880 system (Carl Zeiss) equipped with an oil immersion Plan-Apochromat 63×/1.40 Oil Corr M27 objective, with the incubator set to 37 °C and 5% CO2. The following excitation lasers, used in combination with either confocal or Airyscan acquisition modes, were employed: a 405 nm diode laser for nuclear staining and a 488 nm argon laser for DiO-liposomes. Laser powers were optimized for each sample. All acquired or reconstructed images were processed and visualized using ImageJ (Fiji). For signal quantification, image files were converted to TIFF format. The images were then processed by performing background subtraction, applying automatic thresholding, and analysing nuclear localization using the "Analyse Particles" tool.

Orthogonal views were used in stack mode to visualize all axes and confirm nuclear localization in each cell. Additionally, the images were analysed along the XY and Z axes to assess the spatial distribution of the signal. XY-axis analysis was used to measure two-dimensional (2D) spatial features, including the size and shape of the nuclei, as well as intensity distribution across the focal plane. Z-axis analysis was conducted to investigate the three-dimensional (3D) structure of the nuclei and to assess the depth and volume of the signal in relation to the focal plane. Z-stack images were processed to generate 3D reconstructions, which facilitated the assessment of nuclear morphology and the positioning of the signal within the Z-dimension.

RNA and miRNA Extraction

Following treatment with lipoplexes, primary cells or 3D skin models were processed for RNA and miRNA extraction using the RNeasy Mini Kit (Qiagen, Basel, Switzerland), a widely established and reliable method for isolating high-quality total RNA, which includes both mRNA and miRNA.

The procedure began with the addition of 700 µL of QIAzol reagent, which was used to lyse the cells or organoids. This reagent helps break down cellular membranes and stabilize RNA, including small RNA species like miRNA. To further separate cellular components, 140 µL of chloroform was added to the lysate. After vigorous mixing, the samples were centrifuged at 4°C for 15 minutes at 12,000 × g. This step separates the lysate into three phases: the upper aqueous phase, which contains the RNA, the interphase, and the organic phase. The aqueous phase, containing RNA and miRNA, was carefully collected for further purification.

RNA and miRNA were then purified using the RNeasy mini column, which employs a silica membrane for RNA binding, followed by a series of wash steps to remove contaminants. To maximize RNA concentration and quality, we optimized the elution volumes, ensuring that we obtained high-quality RNA suitable for downstream analyses.

The integrity and quantity of both RNA and miRNA are critical for reliable and reproducible downstream analyses, such as gene expression profiling. Therefore, samples were assessed for quality using the Bioanalyzer PicoChip system (ThermoFisher), which provides precise measurements of RNA integrity.

RNA and miRNA gene expression

For miRNA expression analysis, we used the MicroRNA Reverse Transcription Kit (Applied Biosystems, Foster City, CA, USA), which allows for the efficient reverse transcription of miRNAs into cDNA for subsequent quantification. For RNA gene expression analysis, the High-Capacity cDNA Reverse Transcription Kit (Applied Biosystems) was utilized, following the manufacturer's protocol. Both kits ensure high efficiency and sensitivity in converting RNA into cDNA for accurate downstream analysis.
qPCR was performed on a 7000 ABI Thermofisher (Applied Biosystems, MA, USA) using a TaqMan gene ex-pression assay (FAM dye labeled MGB probe (Applied Biosystems, MA, USA) and the gene-specific primers and probes are shown in Table S2. After qPCR, relative expression was determined using the data from the real-time cycler and the ΔΔCT method. Differences in relative mRNA expression of target genes between control and therapy groups were expressed as fold-changes

Evaluation of immunofluorescence images

Immunofluorescence results were evaluated on blinded specimens by two independent dermatopathologists from the Vall d'Hebron pathology unit. Positive cells per millimetre were quantified using computer-assisted image 4 analysis software (ImageJ 1.45, National Institutes of Health, Bethesda, MD, USA). The staining of the epidermis, dermis and inflammatory infiltrate was evaluated semi- quantitatively using the following blinded score: 0 (<10% positive cells), 0.5 (10-20% positive cells), 1 (20-40% positive cells), 1.5 (40-60% positive cells), 2 (60-80% positives cells), 2.5 (80-90% positives cells) or 3 (>90% positive cells).

2. Supplementary Figures

Supplementary Figure S1. Dose-dependent cytotoxicity of DDC642 in HEKa cells and PBMCs. Cell viability was evaluated following treatment with increasing concentrations of DDC642 (5–40 µg/mL). The percentage of cell viability was determined using the MTT assay, based on fluorescence measurements. Both cell types showed comparable sensitivity to DDC642, with reduced viability at higher concentrations. Experiments were performed in triplicate.

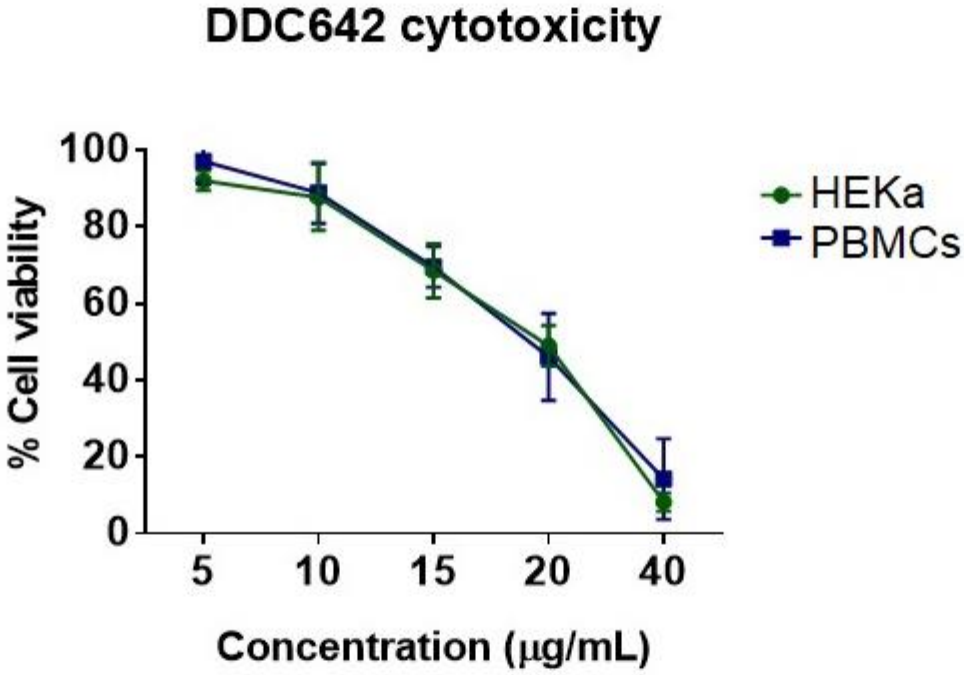

Supplementary Figure S2. Gene expression of additional target genes in keratinocytes treated with lipoplexes at 16:1 and 10:1 ratios. (A) No significant differences were observed across groups for the expression of STK40 and PPP6c when treated with pre-miR-885-5p lipoplex. **(B)** Similarly, for the expression of PSMB5 and TRAF1, no significant changes were detected using anti-miR-31 lipoplex. Data are represented as individual plots from five independent experiments, displayed within the box plot. Control lipoplex containing scrambled miRNAs

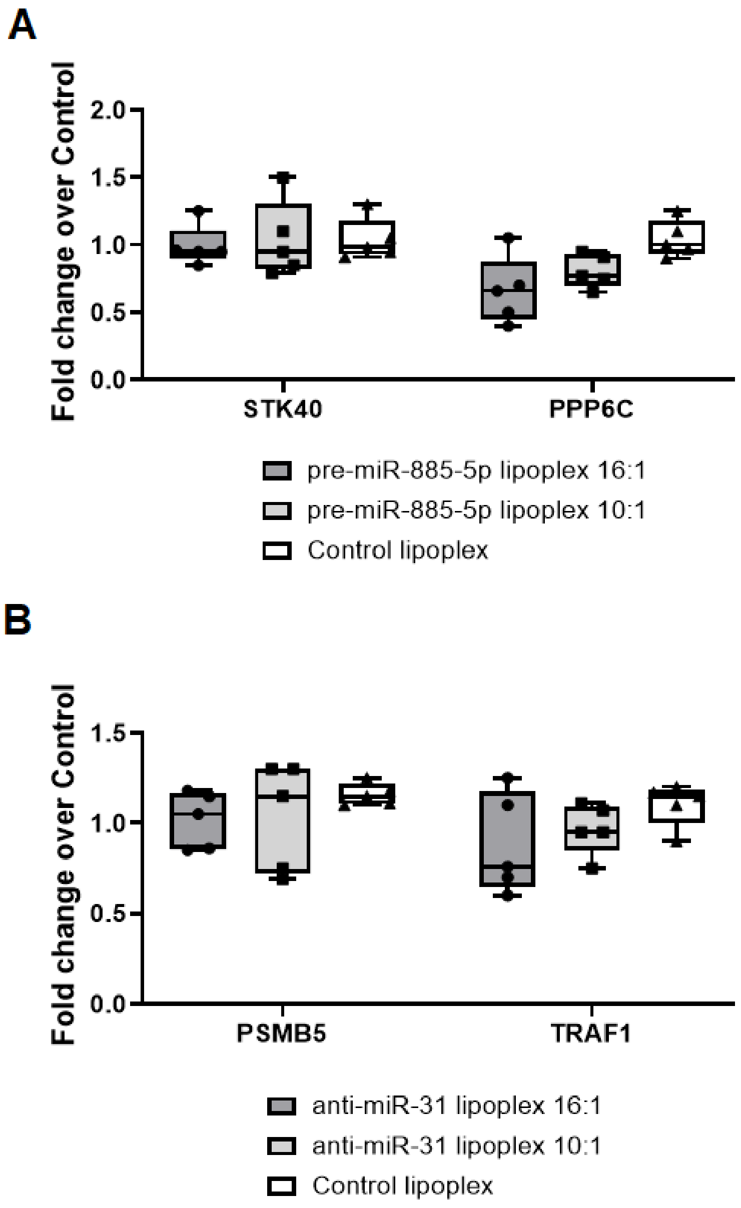

Supplementary Figure S3. Gene expression of NFKB1 in UVB-stimulated primary keratinocytes treated with lipoplexes at a 16:1 ratio. No significant differences were observed in NF-κB expression across groups. Data are expressed as mean ± standard error of the mean (SEM) for five experiments. Statistical analysis was conducted using one-way test comparing lipoplexes to the control.

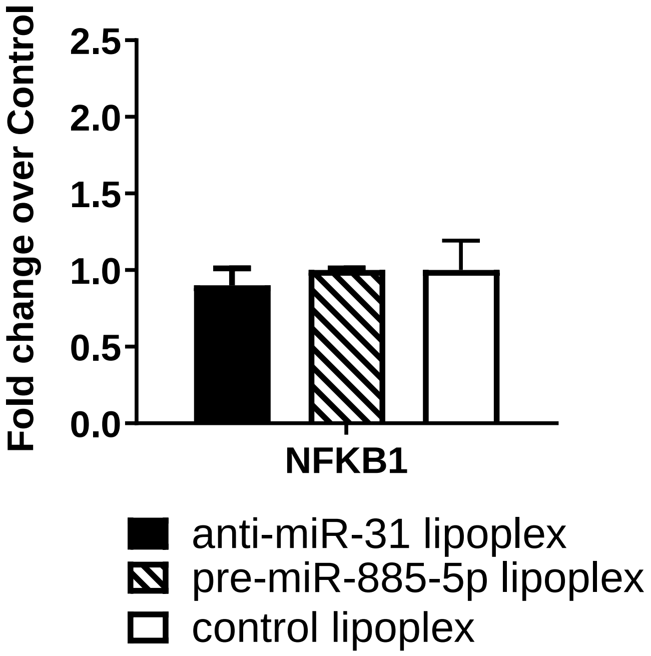

Supplementary Figure S4. Flow cytometric analysis of CD69⁺CD3⁺ T cells in non-stimulated PBMCs. Under non-stimulated conditions, no significant differences were observed between control and anti-miR-485-3p lipoplex treatments, with similar percentages of CD69⁺CD3⁺ T cells across both conditions. Representative flow cytometry plots and quantification of the percentage of CD69⁺CD3⁺ T cells are shown. Data are presented as individual dots within a box plot from three independent replicates. Statistical analysis was performed using Student's t-test, with no significant differences detected (ns).

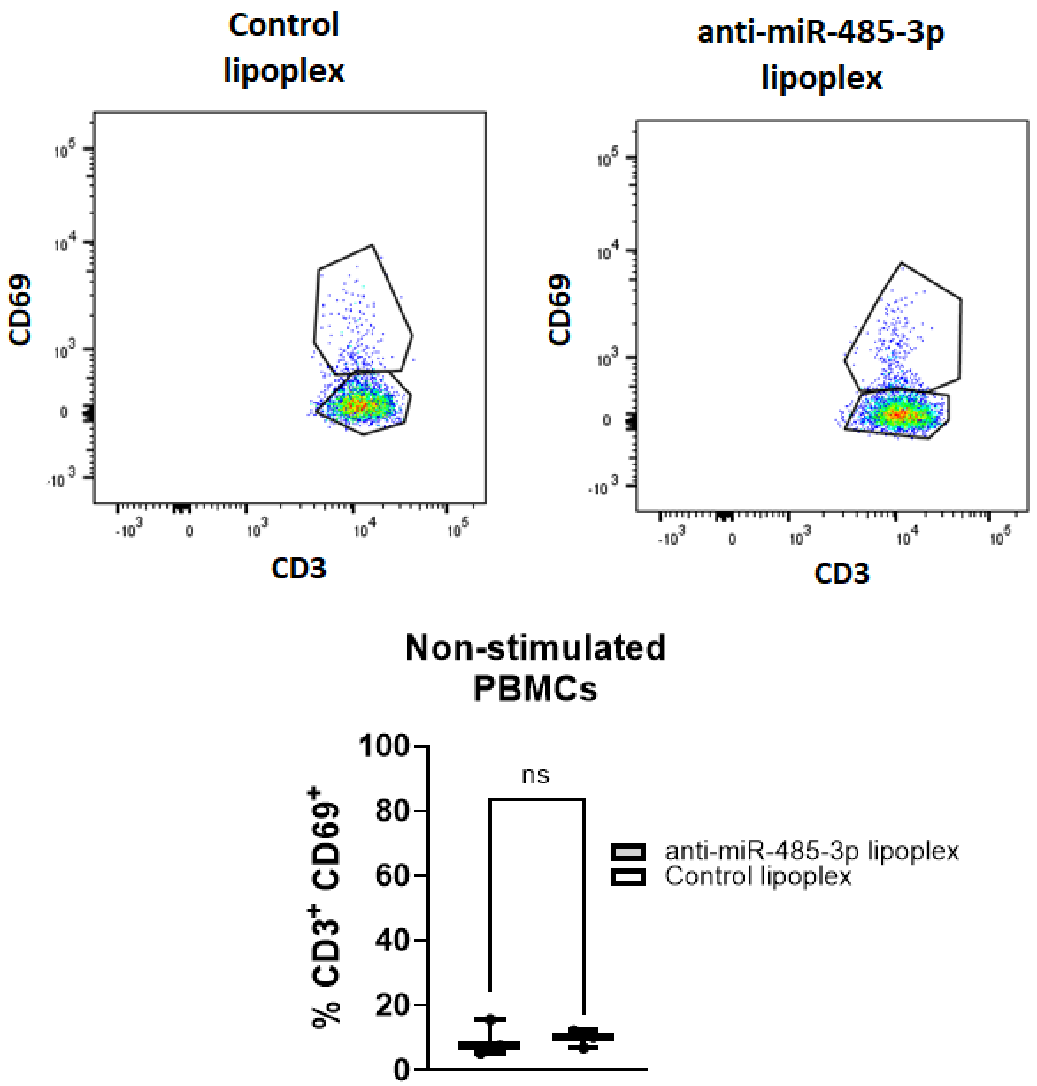

Supplementary Figure S5. Gene expression in immune 3D-skin CLE model after being treatment with anti-miR-485-3p lipoplexes. miRNA and mRNA target gene expression in a skin organoid model following treatment with anti-miR-485-3p lipoplexes (black bars) compared to the control anti-miR lipoplexes (white bars). Genes analyzed include miR-485-3p, PIK3, PRKCD, ICOS and ZAP70. No significant differences (nd) were observed between the groups, as determined using the one-way ANOVA test. Data are expressed as fold change over control, represented as mean ± standard error of the mean (SEM).

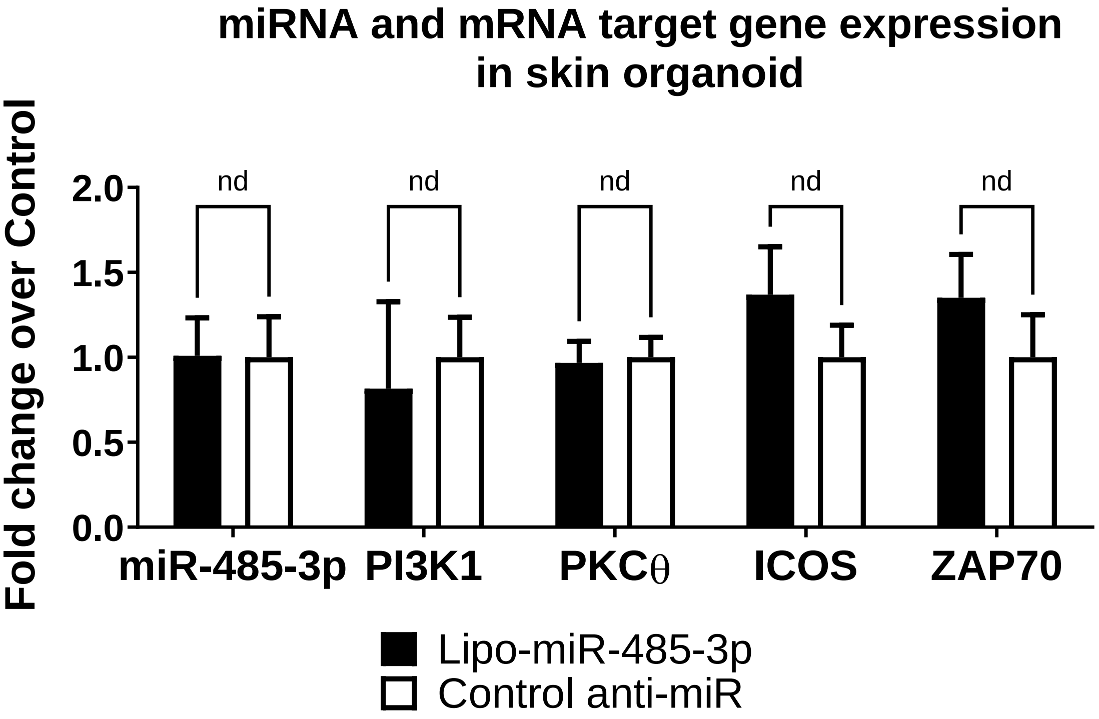

3. Supplementary Tables

Table S1. Clinical and laboratory characteristics of the study subjects.

	**CLE (n=5)**
AGE, mean (SD), yrs	44 (5.1)
Female, n (%)	5 (100%)
Photosensitivity, n (%)	5 (100%)
Smoking, n (%)	2 (40%)
CLASI ACTIVITY, mean (SD)	6.7 (1.8)
CLASI DAMAGE, mean (SD)	0.50 (0.15)
Type of CLE (DLE/CLE)	2/3
Systemic Lupus Erythematosus	1 (20%)
Duration of cutaneous lesions, months, mean (SD)	6.5 (4.6)
ANA positive, n (%)	4 (80%)
Anti-Ro positive, n (%)	3 (60%)

Values are number of patients and between brackets the percent of total patiens. The other values are means ± SD. CLASI: Cutaneous Lupus Erythematosus Disease Area and Severity Index; CLE: cutaneous lupus erythematosus; DLE: discoid lupus erythematosus; SCLE: subacute cutaneous lupus erythematosus; ANA: antinuclear Antibodies; Anti-Ro: autoantibody Ro protein.

Table S2. Primer IDs used in Taqman RT-qPCR from Applied Biosystems.

miRNAs	Assay ID (TaqMan)
**U6 snRNA**	001973
**hsa-miR-31-5p**	002279
**hsa-miR-485-3p**	001277
**hsa-miR-885-5p**	002296

Gene	Assay ID (TaqMan)
**GADPH**	Hs02786624_g1
**STK40**	Hs00894269_m1
**PPP6C**	Hs00254827_m1
**PSBM5**	Hs00605652_m1
**TRAF1**	Hs01090170_m1
**NFKB1**	Hs00765730_m1
**PIK3CA**	Hs00907957_m1
**PRKCD**	Hs01090047_m1
**ICOS**	Hs00359999_m1
**ZAP70**	Hs00896345_m1

Table S3. Antibodies used for immunofluorescence staining.

Primary Antibody	Supplier	Code
**Anti-NFKB**	Genetex	GTX102090
**Anti-PSBM5**	Genetex	GXT104687
**Anti-TRAF1**	Genetex	GXT102372
**Secondary Antibody**	**Supplier**	**Code**
**Alexa-488-conjugated anti-rabbit IgG**	Abcam	ab150077
